# Masked smoothing using separable kernels for CT perfusion images

**DOI:** 10.1186/1471-2342-14-28

**Published:** 2014-08-21

**Authors:** David S Wack, Kenneth V Snyder, Kevin F Seals, Adnan H Siddiqui

**Affiliations:** 1 Dept. of Nuclear Medicine and Center for Positron Emission Tomography, The University at Buffalo, State University of New York, Buffalo, NY, USA; 2 Dept. of Neurosurgery and Toshiba Stroke and Vascular Research Center, The University at Buffalo, State University of New York, Buffalo, NY, USA; 3 School of Medicine, The University at Buffalo, State University of New York, Buffalo, NY, USA

## Abstract

**Background:**

CT perfusion images have a high contrast ratio between voxels representing different anatomy, such as tissue or vessels, which makes image segmentation of tissue and vascular regions relatively easy. However, grey and white matter tissue regions have relatively low values and can suffer from poor signal to noise ratios. While smoothing can improve the image quality of the tissue regions, the inclusion of much higher valued vascular voxels can skew the tissue values. It is thus desirable to smooth tissue voxels separately from other voxel types, as has been previously implemented using mean filter kernels. We created a novel Masked Smoothing method that performs Gaussian smoothing restricted to tissue voxels. Unlike previous methods, it is implemented as a combination of separable kernels and is therefore fast enough to consider for clinical work, even for large kernel sizes.

**Methods:**

We compare our Masked Smoothing method to alternatives using Gaussian smoothing on an unaltered image volume and Gaussian smoothing on an image volume with vascular voxels set to zero. Each method was tested on simulation data, collected phantom data, and CT perfusion data sets. We then examined tissue voxels for bias and noise reduction.

**Results:**

Simulation and phantom experiments demonstrate that Masked Smoothing does not bias the underlying tissue value, whereas the other smoothing methods create significant bias. Furthermore, using actual CT perfusion data, we demonstrate significant differences in the calculated CBF and CBV values dependent on the smoothing method used.

**Conclusion:**

The Masked Smoothing is fast enough to allow eventual clinical usage and can remove the bias of tissue voxel values that neighbor blood vessels. Conversely, the other Gaussian smoothing methods introduced significant bias to the tissue voxels.

## Background

CT perfusion imaging uses many high resolution scans in a dynamic series to determine parametric image maps of Cerebral Blood Flow (CBF), Cerebral Blood Volume (CBV), and Time to Peak (TTP), among other data types. A characteristic of CT image volumes is the high contrast ratio of voxel intensity values located in skull (or calcified regions) versus tissue regions, which can exceed 15:1. Furthermore, with the injection of a tracer, voxels representing vascular regions may have intensity values greater than four times higher than neighboring tissue regions. Kudo et al. demonstrated that the inclusion of vascular voxels could overestimate CBF [1]. The SNR within tissue regions is relatively low. Spatial smoothing is often applied to trade high spatial resolution for improved SNR characteristics. However, regular smoothing overestimates many tissue voxels due to nearby, high-valued vascular voxels.

While the importance of smoothing has been noted in the literature, it usually receives little discussion [2-4]. Klotz and König gave a brief but important description of their smoothing method as a “running mean smoothing procedure that operates separately on brain and vascular pixels” [5] (pg 173). As such, their approach operated in 2D and avoided blurring from smoothing high valued vascular pixels into tissue regions. Our method also operates separately on brain and vascular pixels, however we use a Gaussian kernel. Furthermore, our Masked Smoothing method can execute quickly, even when applied as 3D, by utilizing a combination of separable kernels. This offers an improvement in execution time of a few orders of magnitude relative to what could be achieved otherwise.

A “separable” 3D smoothing kernel can be expressed as the outer product of three vectors, and 3D smoothing can be applied as three successive 1D smoothings in the x, y, and z directions. While Gaussian kernels and mean kernels are separable, they do not remain separable if they must exclude vascular voxels. Our method overcomes this hurdle.

### Related methods

Many smoothing methods are “adaptive” [6] and arrive at an optimal solution through the progressive refinement of an initial solution. Some methods preserve edges [7,8], similar to our desire to separate vessel and tissue voxels. Other methods consider the first or second spatial derivatives [9-12] or use the Discreet Cosine Transform [13]. A strength is these methods do not need a priori knowledge, such as voxel classifications [8]. A 4D extension of bilateral filters varies the weight of neighboring voxels according to distance and intensity, or “similarity” differences [14,15], and has been applied to CT perfusion scans [16]. The TIPS (Time Intensity Profile Similarity) bilateral filter method [17] calculates the similarity of neighboring pixels across all image frames. While this reduces processing time to some degree, the TIPS bilateral smoothing kernel is not strictly separable. While TIPS offers great flexibility in expressing the smoothing formulation, its execution time [17], even applied as a 2D filter, is much slower than what can be achieved using separable 3D kernels.

There are two advantages of CT perfusion imaging over most other image smoothing problems. First, there are multiple image volumes such that voxels in the same spatial location will have the same classification. The second is that there are extreme voxel intensity differences between voxels of different classifications for some image volumes. While thresholding the mean image and the difference of the maximum and minimum images is a simple but powerful way of identifying vascular voxels, more sophisticated methods have been presented for the identification of arteries and veins [18]. Hence Masked Smoothing makes use of the easy access to a mask image of the tissue regions that adaptive smoothing or bilateral filters fail to utilize.

### Masked smoothing algorithm

Smoothing methods typically use a weighted sum of voxels within the smoothing neighborhood of a given tissue voxel, V_x_, to assign a new value to V_x_. The weights are all nonnegative and sum to one. The smoothing neighborhood for a given tissue voxel will, in general, include voxels of different segmentation classes--such as a high valued vessel voxel. This inclusion will have a tendency to artificially increase the smoothed value found at V_x_ from the true underlying tissue value. Our goal is to apply smoothing by only using voxels of like classes. Excluding voxels of a different class could be achieved by setting their weight values to zero, while rescaling the weights of same class voxels so they sum to one.

We define sum of weights (SW) for V_x_ as the sum of all weights of voxels that are both within the area of the smoothing kernel and the mask of the same tissue region (without rescaling). SW will equal one if the all the voxels within the smoothing neighborhood of V_x_ are all of the same class as V_x_. Otherwise SW will be less than one. The reciprocal of SW (1/SW) can be used to rescale the weights so that they sum to one.

Setting some weights to zero and rescaling the remaining weights associated with each voxel within the smoothing neighborhood of Vx is computationally cumbersome. We can simplify the computation by making two changes: 1) Rather than resetting the smoothing kernel weight values of voxels outside of our tissue mask to zero, we instead set voxel values outside of our tissue mask to zero. 2) Rather than rescaling the individual weights within our mask by 1/SW, we rescale the weighted sum of voxel values by (1/SW), employing the distributive property. That is, if SW is known for each voxel, then smoothing the image with non-tissue voxels set to zero and dividing voxel by voxel by SW will result in the desired with-in class masked smoothing. Post-smoothing, non-tissue voxels can be set to zero, replaced by their original values, or smoothed separately.

Fortunately, calculating SW for each tissue voxel is easy. *SW for each tissue voxel is the result of applying the smoothing kernel to the binary mask that designates voxels classified as tissue with a 1 and non-tissue voxels with 0.* This is true since the within-tissue class weights get multiplied by the mask image value of one, whereas the weights for voxels outside our mask are multiplied by zero. Smoothing the binary image is then simply the sum of the weights that are within class, i.e. SW. While we made the above argument for voxels classified as tissue, the same argument can be made in general for any classification.

To summarize, the smoothing process for a given image, Im_orig_, is: 1) create an image mask, Msk, with 1 values at voxel locations representing tissue, and 0 otherwise; 2) Create Im_masked_ by setting all non-tissue voxels of Im_orig_ to zero; 3) Apply the desired smoothing to Im_masked_ and Msk, creating Sm(Im_masked_) and Sm (Msk); 4) Create the “Masked Smoothing” image by setting tissue voxels to the voxel-wise quotient Sm(Im_masked_)/Sm (Msk), and non-tissue voxels to their original values. By using a separable smoothing kernel in Step 3) the Masked Smoothing method will be orders of magnitude faster than directly using the 3D kernel for the calculation.

### Masked smoothing assertions

We developed Masked Smoothing as an alternative to basic Gaussian smoothing, which we term “Simple Smoothing”, and a method where the vessel voxels are set to zero prior to smoothing in an attempt to minimize the impact on tissue voxels, which we term “Removed Smoothing”. We believe that if Simple Smoothing, Removed Smoothing, and Masked Smoothing are used to process the same image, then tissue voxels that neighbor vessel voxels will best maintain their true value with Masked Smoothing. Furthermore, we believe that the differences between smoothing methods can lead to meaningful consequences in the determination of critical CT perfusion parameters. That is, if each smoothing method is applied to the individual time frames of a CT perfusion scan, then tissue voxels that are located near vessel voxels will have significant and meaningful differences in the resulting values of CBF, CBV and TTP depending on the smoothing method used.

## Methods

The Masked Smoothing method was tested against two smoothing methods (Simple and Removed Smoothing) that are similar, but which do not limit the smoothing to tissue voxels. The smoothing methods were tested using simulated data, phantom data, and anonymized CT perfusion data from patients. The simulations provide a framework for determining the noise reduction and bias for each method. The phantom data allows us to test for bias using real scanner data. The CT perfusion data from 23 patients allows an assessment of the impact of bias caused by smoothing the CT Perfusion time series images on the calculation of CBF, CBV, and TTP.

### Smoothing methods

The three smoothing methods were implemented in Matlab (Mathworks, Natick, MA) using Gaussian smoothing kernels:

1) **Simple Smoothing**: The unmodified image volume is smoothed using a Gaussian kernel.

2) **Removed Smoothing**: The vascular voxels are set to zero, and the image is smoothed as in (1) above.

3) **Masked Smoothing**: First, Simple Smoothing is performed on the binary tissue mask. Second, the voxel-by-voxel ratio of the Removed Smoothing image and the smoothed tissue mask image (i.e. the result of the first step of this method) is returned as the Masked Smoothing image. Voxel values outside of the tissue mask are assigned the original image value, except for the phantom experiment. We used this case to also demonstrate that Masked Smoothing can be used to separately smooth both the object and object background.

### Simulation experiment

#### Parameters

We created a simulated volume that included a tissue region; a long thin vascular region, which could be varied in intensity and width; and a border that was set to zero. The dimension of the simulated volume was 100×100×100, and used voxel sizes of 0.4 × 0.4 × 0.4 mm. Each simulation used the following parameters:

1) **Intensity ratio:** The intensity ratio is the ratio between the value assigned to vascular voxels and the tissue region. The tissue value was set to 50. The simulations used intensity ratios of: 2 to 1, 3 to 1, and 4 to 1, which correspond to vascular voxel values of 100, 150, and 200.

2) **SNR:** Gaussian noise was added to all simulation iterations. The standard deviation for the noise generator was set to 50, 25, and ~16.6, which corresponded to SNR values 1, 2, and 3 (lowest noise).

3) **Vessel width:** Is the cross-sectional width of the vascular region. Values used were: .8, 1.6, 2.4 mm.

4) **Smoothing kernel --FWHM**: The isotropic Gaussian smoothing kernel size was set to Full Width Half Max (FWHM) values of 1, 2, and 4 mm.

#### Iteration

**Figure 1:**
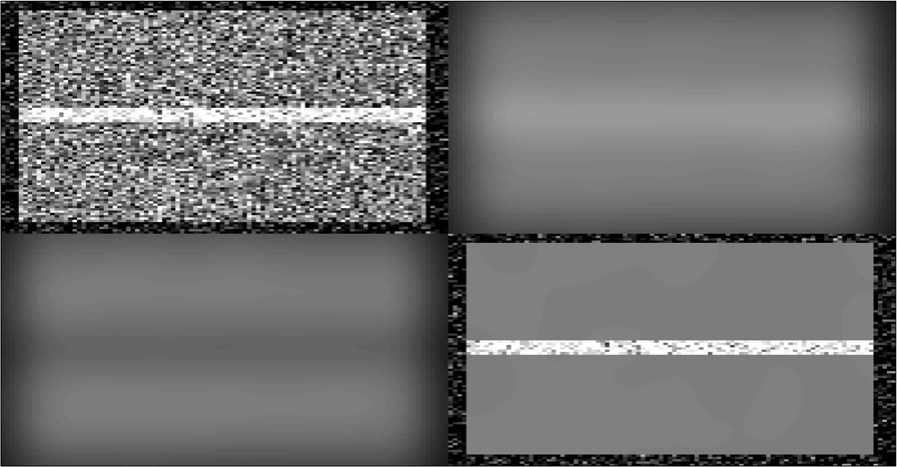
**Simulation vessel with tissue background – raw and smoothed images.** Top Left rectangle is the raw image from the simulation. Top Right rectangle is of Simple Smoothing applied to the raw image. Bottom Left rectangle is of Removed Smoothing applied to the raw image and the Bottom Right rectangle is of Masked Smoothing applied to the raw image. A smoothing kernel of 2 mm was used for each, with a vessel size of 2 mm and a raw image Signal to Noise level of 2.

**Figure 2:**
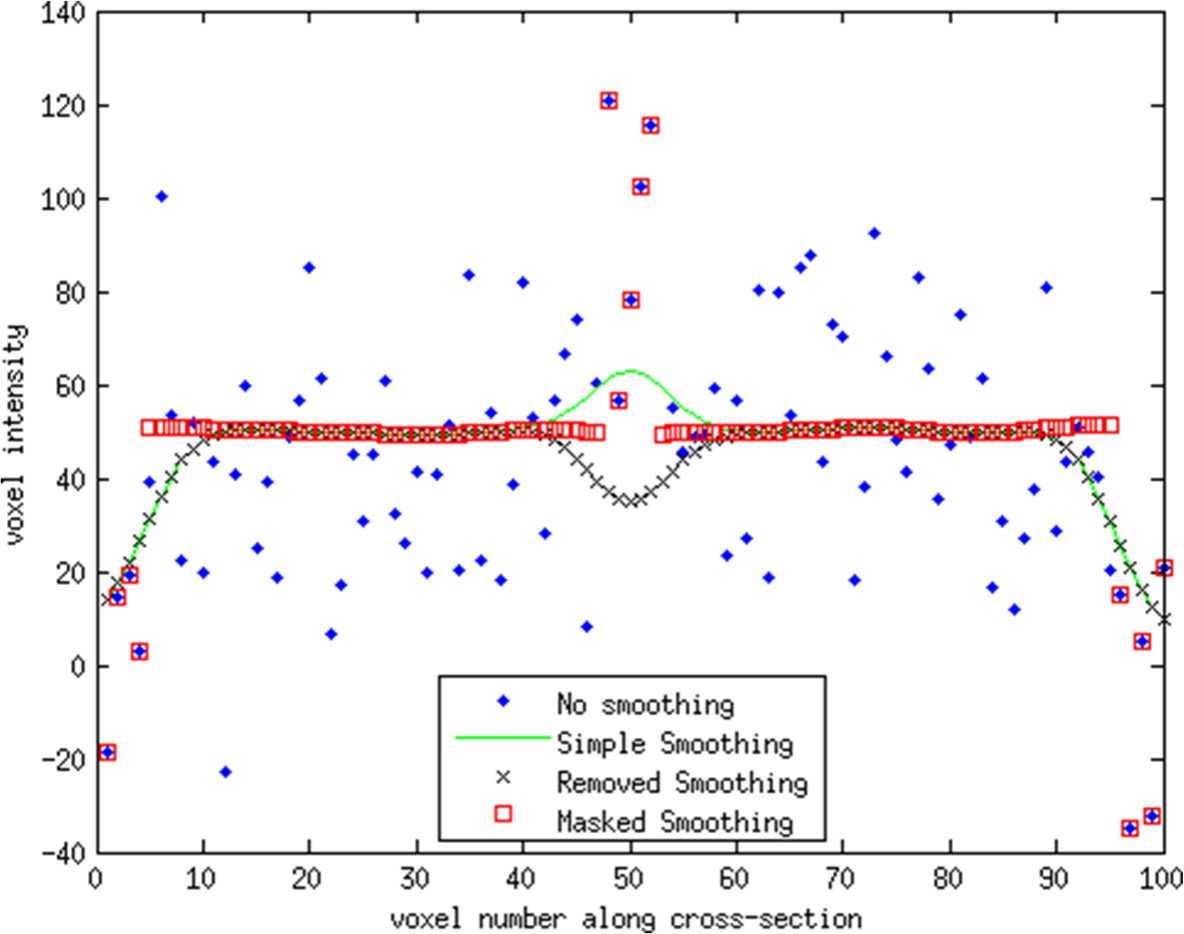
**Vertical line profile for smoothing methods from Figure**1**.** The boundary and vessel voxels are identical for the Masked Smoothing and original values, since Masked Smoothing was only applied to the tissue values. The Simple and Removed smoothing methods had identical results except near the vessel voxels. The Simple Smoothing method over estimates the true value of the tissue near the vessel and underestimates the true value near the boundary. The Removed Smoothing methods underestimates the true value both near the boundary and vessel. For points away from both the boundary and vessel the three smoothing methods gave identical results. The smoothing kernel applied was 2 x 2 x 2 mm for all methods.

For each iteration of a simulation run:

1) The simulated volume was formed with intensity values of tissue voxels set to 50. Vascular voxels were selected according to “Vessel Width”, and assigned an intensity value according to the variable “Intensity Ratio”.2) Gaussian noise was added at a level determined by the Signal to Noise Ratio (SNR), Figure 1, image a.3) All three smoothing methods were applied. All methods used the same Gaussian kernel with the kernel size determined from the variable “Kernel FWHM”, Figure 1, images b-d.4) The value at a tissue location located midway along, and directly next to, the vascular voxels was selected and the value with noise and smoothing applied was recorded. Figure 2 shows the intensity profile for the different smoothing methods for a vertical line passing through the images of Figure 1.

#### Assessment

500 iterations were used to determine the mean and standard deviation for a selected tissue voxel that neighbored the vessel voxels for different settings of parameters. Since in-tissue values in all cases were set to 50, the calculated mean value from 500 iterations even if a high level of noise is added is expected to be very close to 50 unless a bias is present. The four parameters (Intensity Ratio, SNR, Vessel Width, and Smoothing Kernel FWHM) were individually varied using the values given above for each. When one parameter was varied the other values were set to default values (Intensity Ratio = 2 to 1, SNR = 2, Vessel Width = 1.6 mm, and FWHM = 2 mm). One simulation run (500 iterations) was performed for each parameter set allowing for examination of deviation from the expected tissue value of 50 for each smoothing method.

#### CT phantom experiment

**Figure 3:**
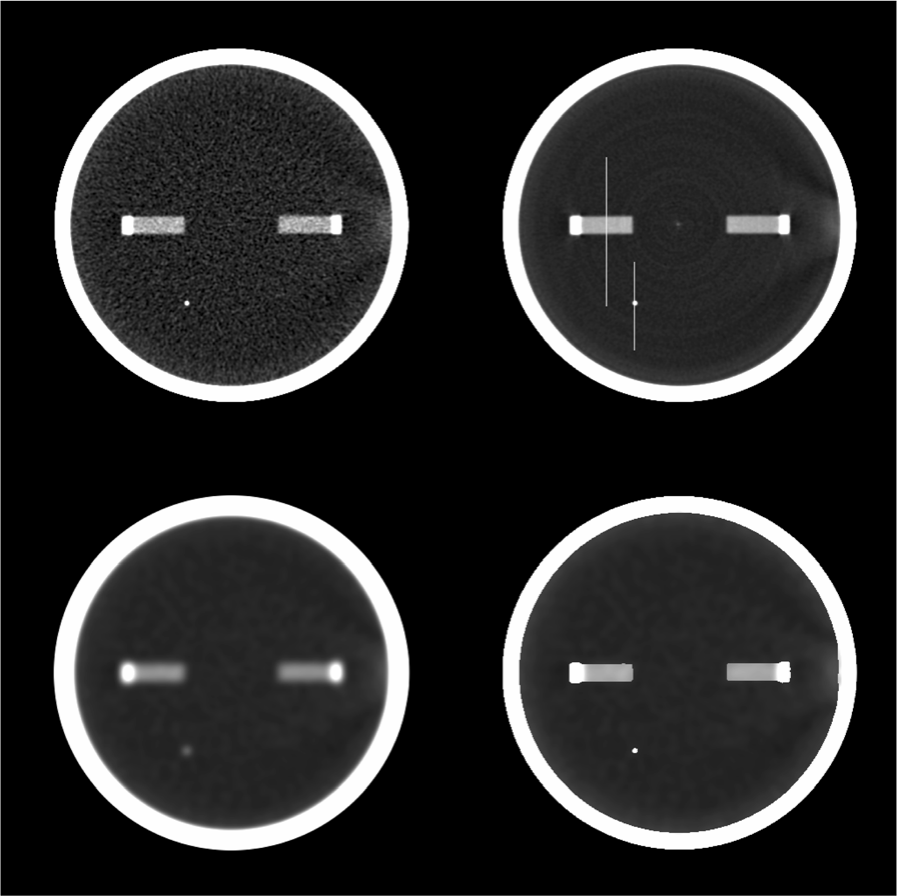
**Image slice from phantom study: raw, mean, and smoothed images.** Top left image is a slice from the first of 30 image volumes. Top right image is the mean of the same slice across all image volumes. Lower left image is the same slice as the upper left but with Gaussian smoothing applied. Lower right image is the same slice as the upper left but with Masked Smoothing applied.

**Figure 4:**
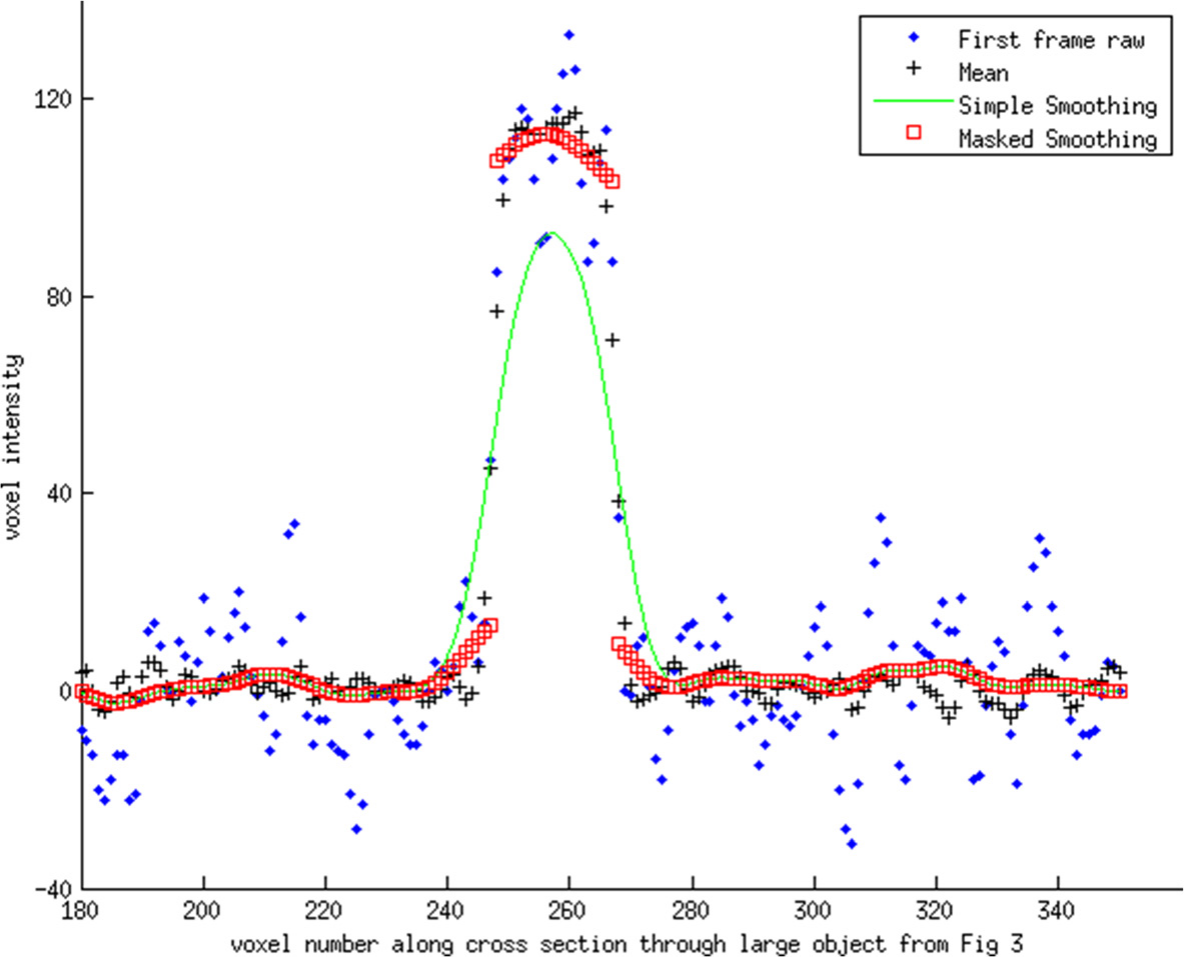
**Intensity profile for line passing through the large object seen in Figure**3**.** Simple Gaussian smoothing underestimates the raw values for the object, but overestimates the values neighboring the object. Values neighboring the object have a small increase in value, which is related to the underlying neighboring voxels being correlated as a result of the reconstruction process. Notice that Masked Smoothing was applied both to the background and the object.

**Figure 5:**
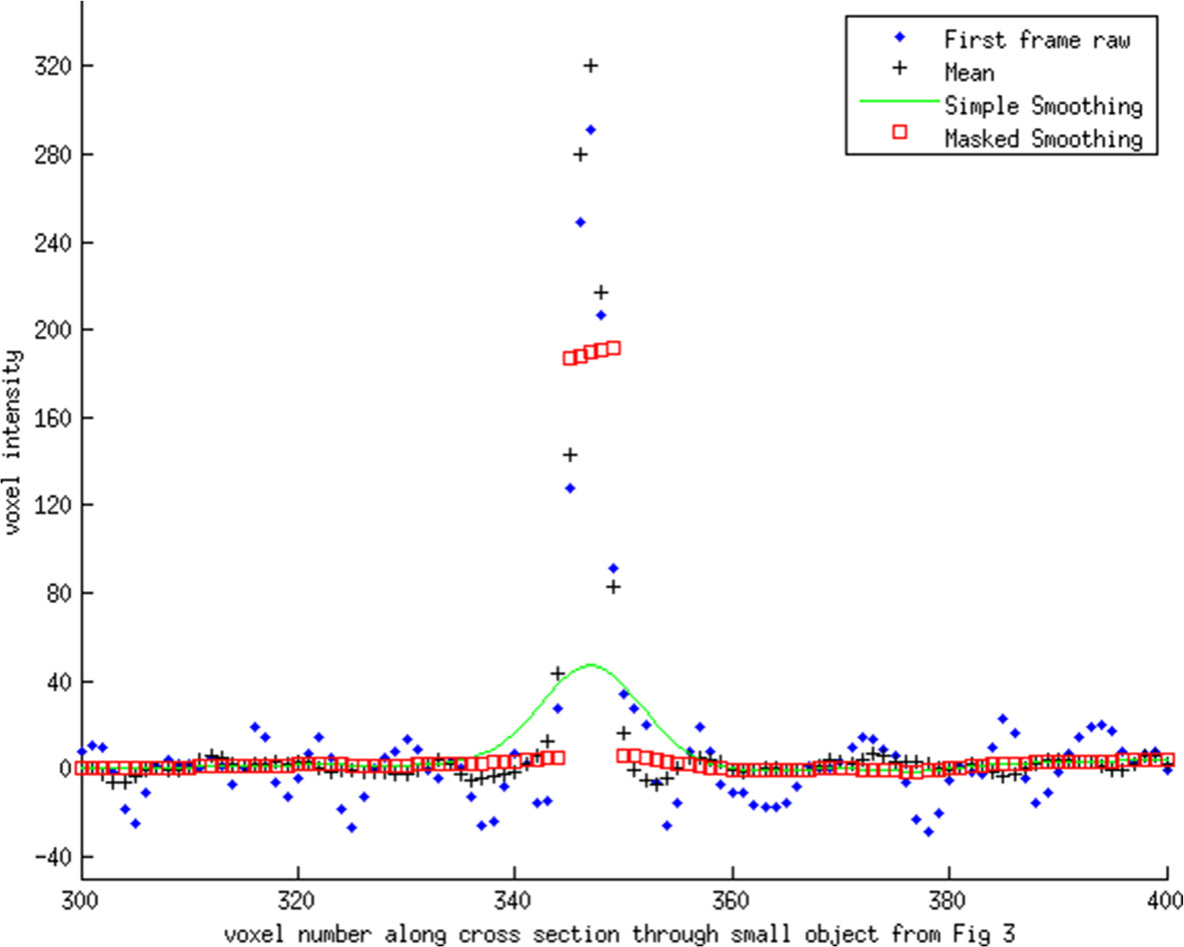
**Intensity profile for line passing through the small object seen in Figure**3**.** The line profile has similar behavior near the object as in Figure 4. Likewise, it can be seen that both smoothing methods give identical results away from the object.

Thirty image volumes of a CT phantom were collected using a Phillips Gemini PET/CT; 0.5 mm thickness, 0 increment, 80 kV, 125 mAs, collimation: 32×1.25 l, rotation time: 0.5 sec, FOV 250, 512 matrix, with voxel size 0.49 × 0.49 × 0.5 mm. All three methods of smoothing, using a 5 × 5 × 5 mm Gaussian kernel, were applied to the first image volume. Additionally, the mean across images volumes was calculated, which was used as the reference because of the reduced noise characteristics. One slice is shown for each smoothing method, and the mean (Figure 3). Two lines were chosen that passed through a large and small object identified on the CT. The line profiles for these lines are shown in Figures 4 and 5. Finally a line of 41 pixels in length was identified directly above of the larger object. The mean and standard deviation was calculated for this set of voxels across all 30 image volumes (i.e. 30 × 41 voxels), for the Simple and Masked smoothing methods, and compared to the mean and standard deviation calculated from the 41 voxels of the mean image. To demonstrate a variation from the simulation experiment, we performed masked smoothing, separately, to both the non-object region and object region.

#### Influence of smoothing method on CBF, CBV, and TTP values

Twenty-three CT perfusion studies were selected from the Neurosurgery department’s stroke research database, at the University at Buffalo. Each dataset consisted of nineteen CT perfusion volumes from a Toshiba Aquilion ONE, 320 slice scanner (with voxel sizes of .42×.42×.5 mm) which were collected from patients presenting with symptoms of a stroke. Images were converted from Dicom to NifTI format, and corrected for motion using SPM8 (http://www.fil.ion.ucl.ac.uk/spm). Image volumes were “skull striped”, and vascular voxels were identified using in-house software written in Matlab. The middle cerebral artery was automatically identified and a center portion was segmented and used for the arterial input function. A similar procedure was used to select the sagittal sinus, and these values were used to ensure the proper scaling of the arterial input function. A parametric image of CBF values was calculated using the maximum slope method, while a CBV image was calculated using the integral of tracer activity divided by the integral of arterial activity.

Tissue voxels immediately adjacent to a selected artery were selected by performing a voxel-wise dilation of the voxels representing a selected artery followed by an intersection with the tissue masks resulting in the elimination of the vascular voxels. For each smoothing method we calculated CBF, CBV, and TTP values for the selected neighboring tissue voxels, using a Gaussian kernel size of 4 × 4 × 4 mm, FWHM. Mean and voxel-wise statistics were calculated to examine differences in CBF, CBV, and TTP due to the smoothing method used.

### Ethics

This project is approved by the University at Buffalo Health Sciences Institutional Review Board.

## Results

### Simulation experiment

Figure 2 displays line profiles, each corresponding to a vertical line across each image of Figure 1, to demonstrate the effects of the Simple, Removed, and Masked Smoothing methods.

#### Simulation experiment intensity ratio

Tissue mean and standard deviation results for different Intensity Ratios are provided in Table 1. When the intensity ratio was varied and the other variables were fixed, all smoothing methods provided over a 10 fold decrease of the standard deviation (Table 1). For the Simple Smoothing method the tissue mean increased with an increase in intensity of the neighboring vessel voxels. The Simple Smoothing method has a 100% increase (bias) for the tissue mean for the highest level of vessel voxel intensity (4 to 1). The Removed Smoothing method showed a bias which lowered the value (~28% decrease) and was unaffected by changes in the intensity of neighboring vessel voxels. The Masked Smoothing method did not show any significant bias in the calculation of the mean tissue value. The standard deviation resulting from the Removed Smoothing was slightly lower than the standard deviation of from the Simple and Masked Smoothing approaches.

**Table 1:** Simulation—intensity ratio: mean values and (standard deviation)

**Method\Intensity ratio**	**1.5 to 1**	**2 to 1**	**4 to 1**
Noise – No smoothing	49.7 (25.1)	50.3 (25.0)	49.0 (25.7)
Simple smoothing	58.2 (1.6)	66.3 (1.6)	99.3 (1.7)
Removed smoothing	33.6 (1.3)	33.5 (1.3)	33.5 (1.4)
Masked smoothing	50.0 (1.9)	50.0 (1.9)	50.0 (2.1)

As the ratio of the intensity of the arterial voxels compared to tissue voxels increased, the values from Masked Smoothing and Removed Smoothing held constant. However, the removed smoothing values were significantly reduced compared to their true underlying value of 50. Masked Smoothing values were equal to their true underlying value in all cases. Tissue voxels for the Simple Smoothing method increased with the increase in the arterial ratio.

#### Simulation experiment -- SNR

Tissue mean and standard deviation results for different SNRs are provided in Table 2. The Simple Smoothing method showed an upward bias, while the Removed Smoothing method exhibited a downward bias. The biases were essentially identical for all three noise conditions. The standard deviation decreased with a decrease in the level of noise (increase of SNR) used in the simulation for all smoothing methods. The standard deviation was markedly smaller for all smoothing methods compared to the non-smoothed images. The Masked Smoothing method did not show a bias in the calculation of the mean tissue value.

**Table 2:** Simulation—SNR: mean values and (standard deviation)

**Method\SNR**	**1**	**2**	**3 (least noise)**
Noise – No smoothing	53.6 (49.0)	49.9 (27.1)	50.8 (16.9)
Simple smoothing	66.4 (3.3)	66.4 (1.6)	60.4 (1.1)
Removed smoothing	33.6 (2.6)	33.6 (1.3)	33.6 (0.8)
Masked smoothing	50.0 (3.9)	49.9 (1.9)	50.0 (1.3)

The standard deviation values were lower for low SNR than for high SNR. Standard deviation values were slightly poorer for the Masked Smoothing method, which is explained by fewer voxels being averaged because non-tissue voxels were excluded.

#### Simulation experiment: change of vessel diameter

Tissue mean and standard deviation results for different vessel widths are provided in Table 3. When the width of the simulated vessel increased, the Simple Smoothing method biased the mean tissue value to greater levels, while the Removed Smoothing method biased the mean value to lesser values. The Masked Smoothing method did not show any bias associated with the mean tissue value. All smoothing methods greatly reduced the standard deviation of the results.

**Table 3:** Simulation—vessel width: mean values and (standard deviation)

**Method\Vessel diameter**	**1**	**2**	**3 mm**
Noise – No smoothing	51.2 (26.0)	50.2 (24.5)	48.5 (25.5)
Simple smoothing	61.3 (1.6)	66.5 (1.7)	68.6 (1.6)
Removed smoothing	38.8 (1.4)	33.6 (1.4)	31.6 (1.3)
Masked smoothing	50.0 (1.8)	50.1 (2.0)	50.1 (2.1)

Masked Smoothing is much closer to the true value of 50 than either of the other smoothing methods. The standard deviation values are markedly better for all smoothing methods than the No Smoothing data. A clear bias is evident for the Simple and Removed Smoothing methods.

#### Simulation experiment: change of smoothing kernel FWHM

The Simple Smoothing method yielded an upward bias for tissue mean values, while the Removed Smoothing method yielded a downward bias. The magnitude of the bias decreased with increased filter size. Masked Smoothing displayed no significant bias of the tissue mean value. For all methods, the standard deviation of the smoothed tissue value decreased when the Smoothing Kernel FWHM increased. Mean and standard deviation values for our three FWHM values and three smoothing methods is provided in Table 4.

**Table 4:** Simulation—smoothing kernel FWHM: mean values and (standard deviation)

**Method\kernel FWHM**	**1**	**2**	**4 mm**
Noise – No smoothing	51.7 (25.1)	49.9 (25.9)	50.0 (24.0)
Simple smoothing	64.6 (4.1)	66.4 (1.6)	60.4 (0.6)
Removed smoothing	35.7 (3.6)	33.6 (1.3)	39.6 (0.5)
Masked smoothing	50.2 (5.1)	50.0 (2.0)	50.0 (0.7)

Only the Masked Smoothing method had mean values close to the true underlying value of 50. Standard deviation values decreased as the kernel size increased.

#### All reported biases

For all simulations the number of iterations was 500, and the standard deviation was relatively small compared to the size of the bias. Hence, all biases reported above were strongly significant (p < 0.0001).

#### Phantom data experiment

The line profiles passing through the small and large object show that each smoothing method is essentially identical for voxels away from the object (Figures 4 and 5). However, where the profiles cross through the object, the Simple Smoothing method has significantly lower valued voxels than the reference, i.e. mean across all image volumes. In contrast, for several voxels on either side of the object, the Simple Smoothing method has higher intensity values than the reference. The Masked Smoothing method has values close to the reference both for voxels located within the object, and outside of the object. We notice that there are a few voxels at either side of the object where the reference values lay between central values for the object and background. This is an indication of the limitation in the scanner resolution and reflects partial volume and an inherent smoothness of the raw data. Similar effects are seen for the line profile passing through the smaller object.

The mean and standard deviation for the 41 voxel line parallel and adjacent to the large object, for all 30 collected image volumes was 6.90 (13.08) HU. With Simple Smoothing, using 5 × 5 × 5 mm Gaussian kernel, the mean increased to 29.78 HU, but the standard deviation decreased to 1.76. With the corresponding Masked Smoothing applied the mean equaled 8.26 HU, i.e. much closer to the original. Further, the standard deviation equaled 1.96 HU, close to same value seen with Simple Smoothing.

#### Patient CT perfusion data – calculation of CBF, CBV, and TTP values

**Figure 6:**
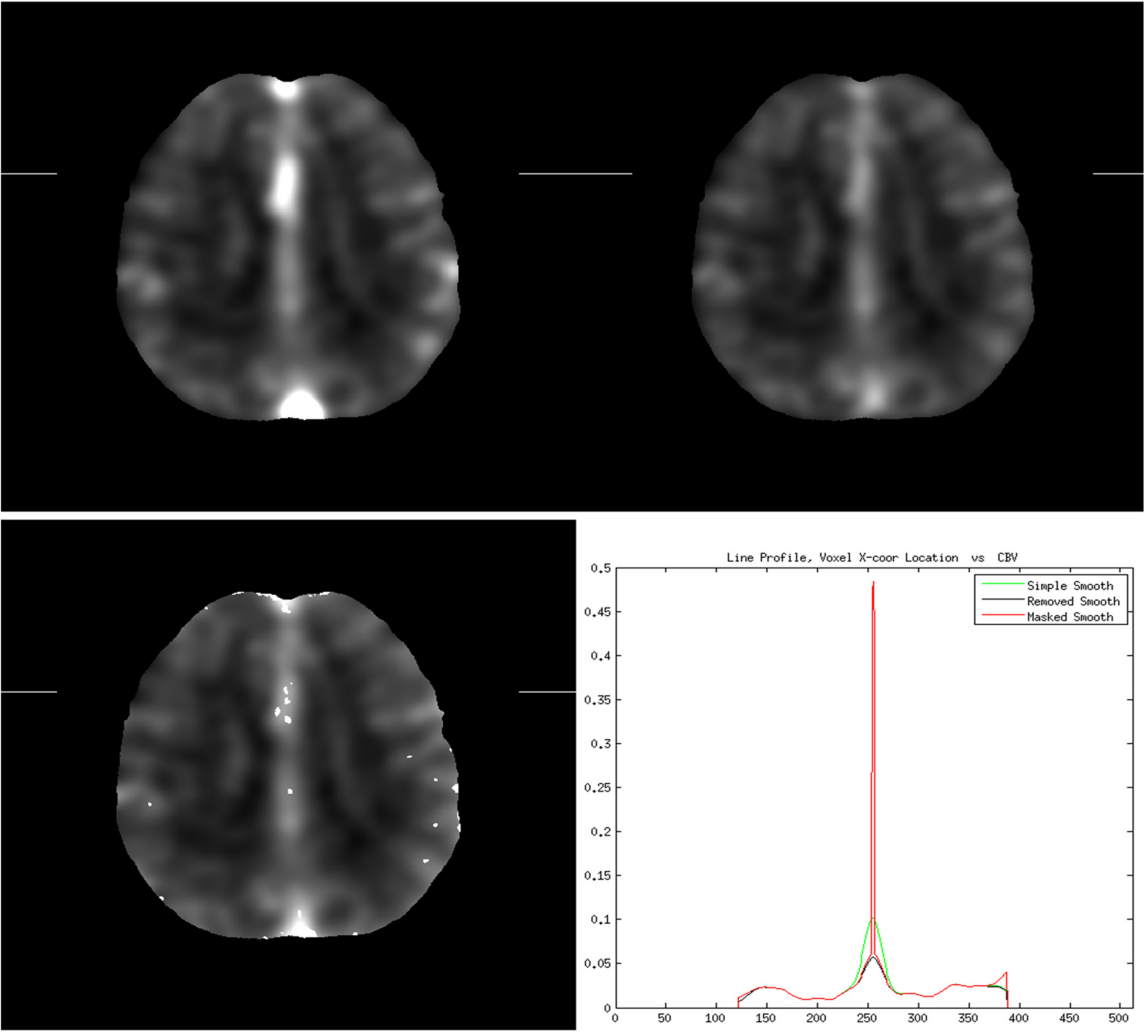
**Simple, Removed, and Masked Smoothing images, with sample line profile.** Top row: Simple and Removed Smoothing images; Bottom row: Masked Smoothing image and the line profile of each along the line connecting the edge marks of the images**.** The line profile of the Masked Smoothing image preserves the high intensity value for a vessel that is crossed near the line center, whereas the peak is much smaller for the Simple Smoothing method, and smallest for the Removed Smoothing method. The Simple Smoothing method has much higher values around the peak, due to the smoothing of the vessel’s intensity into neighboring tissue. Along the right edge the Masked Smoothing method maintained the higher intensity values of the tissue, whereas the Simple and Removed Smoothing methods have lower values that are influenced by surrounding zero values. An 8x8x8 mm smoothing kernel was selected for this illustration to simplify the line profile.

The ROIs of the tissue voxel that neighbored vascular voxels, formed for each of the 23 datasets had a mean size of 376,575 voxels, and was used for determining the mean parameter values. The calculated values for CBF, CBV, and TTP, for the three smoothing methods are reported in Table 5. Mean values for CBV were greater than 50% higher, and CBF were greater than 100% higher, for the Simple Smoothing method than the Masked Smoothing method. Mean values for both CBV and CBF were both more than 30% lower for the Removed Smoothing method than the Masked Smoothing method. The mean TTP values for all smoothing methods were similar.All voxel-by-voxel comparisons for CBF, CBV, and TTP were significantly different (p < < .0001, paired t-test) for all pairwise comparisons of Simple Smoothing, Removed Smoothing, and Masked Smoothing methods, with the exception of TTP calculated from Removed and Masked Smoothing. Identical results (voxel by voxel) were found in comparing TTP calculated from volumes smoothed with the Removed Smoothing and Masked Smoothing methods. Despite finding a significant difference between TTP calculated with Simple Smoothing and either Removed or Masked Smoothing, the magnitude of the difference was very small (1.14 seconds, while the time between volumes was 3 seconds). For illustration, we display the results of the three smoothing methods for one slice using an 8 × 8 × 8 mm kernel (Figure 6).

**Table 5:** Mean Parametric values for subjects’ CT perfusion scan data

**Method\Parameter**	**CBF (ml/(cc x min))**	**CBV (ml/cc)**	**TTP (min)**
Simple smooth	1.48	.112	.52
Removed smooth	.35	.029	.54
Masked smooth	.67	.048	.54

CBF and CBV values were higher and lower than would be expected physiologically for the Simple and Removed Smoothing methods, respectively. Time to Peak (TTP) values were similar for all methods.

Execution time of the Masked Smoothing method was 66 seconds for the 512×512×320×19 voxel CT perfusion image volume using a Gaussian Filter with size 2 × 2 × 2 mm FWHM, which required a 25 × 25 × 21 voxel kernel. Execution time using an 8 × 8 × 8 mm FWHM Gaussian filter, which required a kernel of 99×99×93 voxels, was 81 seconds. Execution time was measured using “tic” and “toc” Matlab functions, on a multi-user Dell PowerEdge R710 server with Dual 2.4 GHz processors, and 48 GB RAM.

## Discussion

Our simulation and phantom data show that the Simple (i.e. ordinary Gaussian smoothing) and Removed Smoothing introduce a significant bias to tissue voxels that neighbor vessels, whereas our Masked Smoothing method did not introduce a bias. Our experiment using patient data revealed that the bias of the Simple and Removed Smoothing methods had a large impact on the calculation of CBF and CBV. The Removed Smoothing method had the lowest values for CBF and CBV and were influenced by factoring in zero values in the place of neighboring vascular values. The Simple Smoothing method had increased CBF and CBV values for tissue voxels that neighbor vessels that were not physiologically reasonable. The Masked Smoothing method had physiologically reasonable values for CBF and CBV, between the extremes returned by the Removed and Simple smoothing methods (Table 5). Given that the Masked Smoothing method does not introduce bias (as opposed to the Removed and Simple Smoothing methods), is easy to implement, and executes fast enough to allow clinical use, we advocate its use over the other methods for the smoothing of CT perfusion images.

### Study design

We used simulated data to test the performance characteristics of the three smoothing methods in situations where the smoothing neighborhood for a tissue voxel included the much higher valued vessel voxels. In all simulations the tissue and vessel intensity values remained constant, allowing us to measure the effect of varying vessel characteristics (both vessel size and tracer concentration), the effect of varying SNR, and influence of the smoothing kernel FWHM. Using phantom imaging we were able to further show potential biases caused by the different smoothing methods. Using real world data from 23 patients, we also compared Simple, Masked, and Removed Smoothing to examine whether the theoretical improvement seen on simulations can have a real life impact in the calculation of CBF, CBV, and TTP. Using this approach we not only showed that Masked Smoothing did not have the bias of the other methods, but we also demonstrated the large practical impact this has on determining physiological parametric images for CBF and CBV.

### Change of intensity ratio/vessel width

Our simulation experiments indicate that the Simple Smoothing method has a large upward bias for tissue voxels surrounding a vessel that increases as the intensity of the vessel voxel increases. By setting the vessel voxels to zero for the purpose of smoothing, the Removed Smoothing method has a downward bias for tissue voxels neighboring a vessel voxel that is both fixed and independent of the vessel voxel’s intensity level. The Masked Smoothing method avoided bias by compensating for voxels set to zero. Increasing the vessel width increased the bias for the Simple Smoothing method, which reflects that a greater number of high intensity vessel voxels are within the smoothing neighborhood of the tissue voxel. The Removed Smoothing method also increased its bias (downward) with an increase in vessel size. This is reasonable, since for a given smoothing neighborhood the Removed Smoothing method would have a greater number of vascular voxels set to zero as the vessel width increases. Again, by compensating for voxels that were set to zero the Masked Smoothing method did not exhibit a bias.

### Change of SNR/smoothing kernel FWHM

All smoothing methods provided a large decrease in the noise level. Increasing the noise level caused an increase in the standard deviation measured for all methods, but had no effect on the calculated mean value. Increasing the filter kernel for all methods reduced the measured standard deviation. Increasing the filter size from 1 mm to 2 mm increased the bias for both Simple and Removed Smoothing. However, increasing the smoothing kernel further to 4 mm resulted in the smallest bias. The change in bias reflects the weighted proportion of voxels that are within the smoothing neighborhood. With the 4 mm smoothing kernel, the smoothing is incorporating a significant number of voxels from the “other-side” of the vessel, hence lessening the influence of the vessel itself. As in all cases, the Masked Smoothing exhibited no significant bias.

### Influence of smoothing method on the calculation of CBF, CBV, and TTP

Smoothing is a critical noise reduction pre-processing step prior to the calculation of physiologic parameters as we have demonstrated previously using simulations [19-21]. The CBF and CBV derived from the Simple and Removed Smoothing methods differed, approximately, by a factor of four for tissue voxels close to vessels, thus demonstrating the critical importance of the smoothing method. The CBF and CBV values, calculated using the Masked Smoothing method, were in-between and significantly different from the other smoothing methods, and closest to physiologically expected values. Since the Masked Smoothing approach showed no bias on the simulated data, we believe these CBF and CBV values are the most accurate. The TTP values for the Removed and Masked Smoothing were identical because the time activity curves for a given voxel will only differ by a scaling multiple, and were close to the Simple Smoothing method.

### Filter selection

We used a Gaussian smoothing kernel for our implementation because it is commonly used for medical images, and allows for fast implementations because it is separable. Our 3D execution times for an entire volume was significantly faster than a 2D TIPs bilateral filter on a single slice. Klotz and König [5] also applied smoothing separately on brain and vascular voxels. Their approach used multiple applications of a mean filter, whereas we utilized a Gaussian kernel. Our approach would also work with mean filters, since they are also separable. There are very fast methods for implementing mean filters; and furthermore, multiple passes of a mean filter can be used to approximate a Gaussian filter. However, internal timings during development favored our approach.

### Segmentation and segmentation

The Masked Smoothing method assumes that satisfactory segmentation is available. However, if the thickness between planes is high then partial volume effects may hinder segmentation. If a vessel voxel were to be classified as a tissue voxel, then neighboring tissue voxels will be biased upward, especially as the tracer concentration peaks in the vessel. However, this bias cannot exceed the bias from using Simple Smoothing. Because of the quantitation, some voxels partially represent both underlying tissue and vessel. This is not a problem in practice. If this voxel is excluded, the estimate for a nearby tissue voxel proceeds without using the value. If the voxel is included, then a neighboring voxel may be biased upward, but the effect will be minimal since the voxel partially represents tissue and thus will not reach especially high intensity levels. This is similar to the situation seen with the phantom data, where the mean of the raw data shows a gradual increase to the higher intensity object. In this case the Masked Smoothing best approximated the best estimate of the true value found by calculating the mean across 30 image volumes. Finally, our method allows both the arterial and tissue regions to be smoothed separately.

## Conclusion

We demonstrated that the Masked Smoothing method executes rapidly and can readily integrate into existing smoothing kernels. The Masked Smoothing method does not introduce a bias in situations where nearby voxels have a different classification and a large difference in intensity values. This accuracy, coupled with speed, gives the Masked Smoothing method the potential to significantly improve the clinical processing of perfusion imaging.

## Competing interests

The authors declare they have no competing interests.

## Authors’ contributions

DSW developed the algorithm and developed software for the experiments. DSW and KFS drafted the manuscript. KFS, KVS, and AHS set criteria for and identified appropriate scans for inclusion. All authors participated in the experimental design, and have read and approved the final manuscript.

## Pre-publication history

The pre-publication history for this paper can be accessed here:

http://www.biomedcentral.com/1471-2342/14/28/prepub
